# Spatial Modelling of Environmental Risk Factors Influencing Schmallenberg Virus Exposure in German Sheep

**DOI:** 10.1155/tbed/7317792

**Published:** 2026-02-20

**Authors:** Frederik Kiene, Hannes Bergmann, Martin Ganter, Benjamin U. Bauer

**Affiliations:** ^1^ Clinic for Swine, Small Ruminants and Forensic Medicine, University of Veterinary Medicine Hannover, Foundation, Hannover, Lower Saxony, Germany, tiho-hannover.de; ^2^ Field Station for Epidemiology (Bakum), University of Veterinary Medicine Hannover, Foundation, Hannover, Lower Saxony, Germany, tiho-hannover.de; ^3^ Friedrich-Loeffler-Institut, Federal Research Institute for Animal Health, Institute of Epidemiology, Südufer 10, Greifswald-Insel Riems, 17493, Mecklenburg Western Pomerania, Germany, fli.de; ^4^ Friedrich-Loeffler-Institut, Federal Research Institute for Animal Health, Institute of Immunology, Südufer 10, Greifswald-Insel Riems, 17493, Mecklenburg Western Pomerania, Germany, fli.de

**Keywords:** cattle density, climate variables, *Culicoides* midges, environmental risk factors, geospatial analysis, land use, Schmallenberg virus, seroprevalence, spatial modelling, vector-borne disease

## Abstract

Schmallenberg virus (SBV) is a *Culicoides*‐borne *Orthobunyavirus* causing congenital malformations and reproductive losses in ruminants, with substantial economic and livestock health impacts across Europe. While outbreaks have been linked to specific climatic and environmental conditions, the drivers of SBV transmission in endemic regions remain poorly defined. It is unclear to what extent spatial variation in SBV seroprevalence reflects environmental risk factors in temperate regions with intensively managed livestock systems such as those in Germany. Spatially explicit generalised additive models (GAMs) and predictive risk mapping are, hence, applied to investigate whether landscape, climate or host availability influence SBV exposure risk in sheep flocks across five German federal states. Serological data were obtained from 70 sheep flocks (*n* = 2723 animals; autumn 2017 to spring 2018) and 69 environmental variables were used in the spatial risk analysis. Environmental heterogeneity showed limited explanatory power for SBV seroprevalence. The final GAM explained 50.6% of deviance and identified cattle density as the strongest positive predictor (odds ratio [OR] = 1.01, *p* < 0.001), while nature reserve coverage (OR = 0.13, *p* = 0.015) and summer temperature during the wettest quarter (OR = 0.95, *p* = 0.021) were negatively associated. No spatial clustering was detected, and the predicted risk surface revealed only modest regional variation. These findings suggest that farm‐level factors and cattle‐associated vector habitats are more relevant to SBV transmission than broader climatic or land use gradients in ecologically uniform settings. The diffuse spatial pattern underscores a general vulnerability of German ruminants to *Culicoides*‐borne viruses and supports the need for targeted surveillance and farm‐focused vector control. This modelling framework may assist in future risk assessments for emerging arboviruses under changing climate and agricultural conditions.

**Summary**

• The prevalence of antibodies against the endemic Schmallenberg virus (SBV) in German sheep flocks is independent from most climate, land use, topographic and host population density factors.
• Sheep flocks from regions with a high density of cattle were significantly more likely to develop high prevalences of antibodies against the SBV.
• Nature reserve area and mean temperature of the wettest quarter were negatively associated with SBV risk, indicating they may be protective factors.
• Spatial predictions reveal a largely uniform and unstructured distribution of SBV seroprevalence, indicating widespread and diffuse exposure risk across the study area.
• The study suggests that small ruminants in Germany are broadly vulnerable to emerging *Culicoides*‐borne viruses.

## 1. Introduction

Haematophagous biting midges of the genus *Culicoides* (family Ceratopogonidae) act as vectors for many viral infectious diseases in wild and domestic ruminants all over the world. In Europe, the bluetongue virus (BTV), the epizootic haemorrhagic disease virus (EHDV) and the Schmallenberg virus (SBV), by far the most important pathogens in this respect, are largely transmitted by identical and widespread native *Culicoides* species [1–5]. During the last 15 years, these *Culicoides*‐borne viruses showed a very dynamic pattern of distribution [5, 6].

SBV, a member of the Simbu serogroup within the genus *Orthobunyavirus*, is closely related to the Akabane, Aino and Shamonda viruses. It first emerged in 2011 in connection with mild fever and loss of productivity in dairy cows near the town of Schmallenberg in North Rhine‐Westphalia, Germany [7–9]. Since its emergence, SBV has spread far beyond Europe and was detected across parts of Africa and Asia [9]. In pregnant ruminants, infection during critical windows of gestation can lead to severe congenital malformations. In sheep and goats, this vulnerable phase falls between days 28 and 56 of pregnancy [10, 11], while in cattle, it occurs between days 70 and 150 [12]. The resulting brain, spinal, jaw and limb deformities can cause complications during birth and usually lead to non‐viable offspring [10, 13, 14]. Embryonic loss is common when infection occurs early in pregnancy. Infection after the vulnerable period of gestation tend to result in the development of fetal immunity with precolostral antibodies. The vast majority of SBV transmission events is attributable to transcutaneous inoculation by infected midges [15, 16]. In domestic ruminants, the viraemic phase, where blood feeding midges can acquire the pathogen, begins 2–4 days after infection and lasts for a very short period of around 4–6 days [10, 17]. Infected sheep and cattle begin to develop IgG antibodies within 10–21 days after infection [13, 17, 18]. Antibody levels then increase for about 4 weeks before stabilising for at least 16 months resulting in protective immunity [19, 20].

Although SBV disease is not categorised in the regulation (EU) 2018/1882 according to the New Animal Health Law of the European Union, positive detections in Germany must be reported in accordance with the national Animal Health Law. Since 2013, the last year with higher reporting frequencies, these reports have been undulating on a low level in the upper double‐digit range [21–24]. However, a recent SBV seroprevalence study in wild ruminants in 2023 found that over 30% of the animals born that year had already developed antibodies, suggesting substantial ongoing circulation [25]. These findings highlight the continued risk of re‐emergence and SBV outbreaks in susceptible host populations.

*Culicoides* vectors in Europe are active from early spring to late autumn [6], possess poor flying abilities [26] and generally remain in close distance to their larval habitats [27]. The development of larvae and pupae is commonly considered to depend on wet or humid conditions [28]. In fact, only a few species directly dependent on stagnant or flowing water [27, 29]. Most species develop in moist organic‐rich topsoil, such as marshes, bogs or swamps [30, 31]. Dung habitats like manure pits in farm environments are also important breeding grounds for many *Culicoides* species [31–35]. For midges of the *Culicoides obsoletus* complex, considered to be the main vectors of SBV, manure was shown to be an important breeding substrate [36, 37].

Environmental factors influencing SBV and other *Culicoides*‐borne disease transmission dynamics remain poorly understood. Gubbins et al. [38] estimated a minimum temperature of 12.3°C for virus replication within the vector. Temperatures above 14°C and altitudes less than 400 m above sea level were identified as risk factors for SBV exposure of sheep and goats in southern Spain [39]. Other risk factors include host‐related attributes such as species differences, female sex and older age [9, 40–45], as well as husbandry and management practices [45–50].

To address current knowledge gaps, this study investigated the influence of environmental factors on SBV seroprevalence in domestic sheep flocks in Germany, specifically assessing climate factors, land use, topography and host population density. Seroprevalence data from sheep sampled in 2017–2018 were spatially analysed in relation to multiple geospatial covariates. Based on known SBV biology, we hypothesised that higher SBV seroprevalence would be observed in regions with warmer and wetter climates, lower altitudes, higher surface water availability and greater host (manure) density. Additionally, due to the limited flight abilities and range of *Culicoides* spp., we expected coastal wind exposure to act as a protective factor. This study aims to improve understanding of the environmental risk landscape for SBV in temperate European climate and support targeted surveillance for emerging *Culicoides*‐borne pathogens in ruminants.

## 2. Materials and Methods

### 2.1. Study Population and Laboratory Analysis

**Figure 1:**
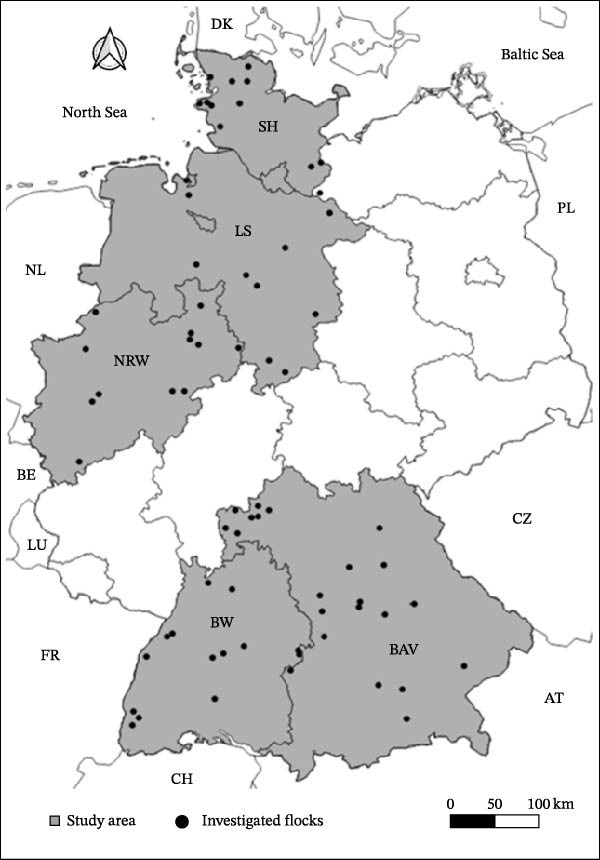
Geographic distribution of 70 sheep flocks sampled across five German federal states and tested for Schmallenberg virus antibodies. States: NRW, North Rhine‐Westphalia; SH, Schleswig‐Holstein; LS, Lower Saxony; BAV, Bavaria; BW, Baden‐Wuerttemberg (modified after Kiene et al. [45]).

This study is based on serum samples from sheep taken between autumn 2017 and spring 2018 as part of a broader investigation into the seroprevalence of *Coxiella burnetii* in German small ruminant populations [51, 52]. It aims to improve understanding of the environmental risk landscape for SBV in the temperate European climates and to support targeted surveillance for emerging *Culicoides*‐borne pathogens in ruminants. A total of 70 flocks of sheep were sampled. Flock sizes ranged from 19 to 3250 individuals. In 27 flocks, goats were also present alongside sheep. The sampled flocks were geographically distributed across five federal states (Schleswig‐Holstein, Lower Saxony, North Rhine‐Westphalia, Baden‐Wuerttemberg and Bavaria). As can be seen in Figure 1, the spatially unified boundaries of these five German federal states define the study area applied in this analysis, constituting a northern and a southern part. The sampling regime on flock level (mean number of samples per flock = 38.9) was designed to estimate a prevalence of 3% with 95% confidence and 5% precision. For the present analysis, an expected SBV prevalence of 50% was assumed, which corresponds to an estimated precision of approximately ±14% for herd‐level prevalence, depending on herd size. As a data basis for this work, as well as for a related study on management‐related risk factors [45], a total of 2723 sheep sera were tested for antibodies against SBV using a commercial ELISA kit (Schmallenberg Virus Competition Multi‐Species, IDvet, Grabels, France). Detailed procedures for sample collection, storage and laboratory testing are provided in Kiene et al. [45]. All 70 investigated sheep flocks included seropositive animals, yielding a between‐flock prevalence of 100%. The mean within‐flock prevalence was 60.7% (*σ* = 16.5%, range: 15.0%–97.3%). Based on the diagnostic sensitivity of the ELISA kit (97.6%, [53]), the estimated true seroprevalence of SBV antibodies among the total sampled sheep population was 61.5% [45].

### 2.2. Environmental Data

A total of 69 environmental parameters of the categories: climate, wind, topography, land use and population density of potential SBV host species were examined for possible effects on the exposure of sheep to SBV and, thus, the development of antibodies. All data on which the environmental parameters are based, were retrieved from openly available data sources. An overview of all the parameters under investigation is provided in Table 1. The processing, analysis and visualisation of geospatial data was performed applying the software QGIS version 3.34.11 (QGIS.org, 2024. QGIS Geographic Information System. QGIS Association. http://www.qgis.org) [58] and R version 4.4.0 [59] using the packages ‘sf’ [60], ‘terra’ [61], ‘exactextractr’ [62] and ‘tmap’ [63].

**Table 1:** Overview of data sources for the 69 studied environmental factors.

Parameter	Definition	Source
Climate variables		DWD Climate Data Center (CDC): Annual Grids, version v1.0; data from 2017
Minimum temperature	Annual grid of monthly averaged daily maximum air temperature (°C)	
Mean temperature	Annual grid of monthly averaged daily mean air temperature (°C)	
Maximum temperature	Annual grid of monthly averaged daily minimum air temperature (°C)	
Sunshine duration	Annual sum of monthly sunshine duration (h)	
Precipitation	Annual sum of monthly precipitation (mm)	
Drought index	Annual grid of the de Martonne drought index	
Snow cover days	Annual grid of number of snow cover days	
Frost days	Annual grid of number of days with a minimum air temperature of <0°C	
Summer days	Annual grid of number of days with a maximum air temperature of ≥25°C	
Hot days	Annual grid of number of days with a maximum air temperature of ≥30°C	
Onset of vegetation	Annual grid of the onset of the vegetation period (day of the year)	
Apple flowering	Annual grid of the flowering day of apple trees (day of the year)	
End of vegetation	Annual grid of the end of the vegetation period (day of the year)	
Wind power density	Annual grid of wind power density in 50 m height	Global Wind Atlas [54]
Bioclimatic variables		www.worldclim.org [55]
BIO1	Grid of annual mean temperature (°C)	
BIO2	Grid of mean diurnal range (°C, mean of monthly [max temp–min temp])	
BIO3	Grid of isothermality (BIO2/BIO7) × 100	
BIO4	Grid of temperature seasonality (standard deviation × 100)	
BIO5	Grid of maximum temperature of the warmest month (°C)	
BIO6	Grid of minimum temperature of the coldest month (°C)	
BIO7	Grid of annual temperature range (°C, BIO5–BIO6)	
BIO8	Grid of mean temperature of the wettest quarter (°C)	
BIO9	Grid of mean temperature of the driest quarter (°C)	
BIO10	Grid of mean temperature of the warmest quarter (°C)	
BIO11	Grid of mean temperature of the coldest quarter (°C)	
BIO12	Grid of annual precipitation (mm)	
BIO13	Grid of precipitation of the wettest month (mm)	
BIO14	Grid of precipitation of the driest month (mm)	
BIO15	Grid of precipitation seasonality (coefficient of variation)	
BIO16	Grid of precipitation of the wettest quarter (mm)	
BIO17	Grid of precipitation of the driest quarter (mm)	
BIO18	Grid of precipitation of the warmest quarter (mm)	
BIO19	Grid of precipitation of the coldest quarter (mm)	
Land use variables		Geofabrik, OpenStreetMap, 2018
Forest	Land cover with managed tree area (%)	
Meadow	Land cover with permanent grassland, mainly for hay or extensive grazing (%)	
Nature reserve	Land cover with protected area, may include diverse habitats (%)	
Residential	Land cover with area for housing: single‐family homes, apartments, etc. (%)	
Cemetery	Land cover with area designated for burial, religious or secular (%)	
Commercial	Land cover with zones for offices, services and hotels; excludes retail stores (%)	
Farmland	Land cover with cultivated land for crops like grains, vegetables (%)	
Farmyard	Land cover with farm buildings and courtyard; excludes cultivated fields (%)	
Grass	Land cover with general grassy area; no specific use implied (%)	
Industrial	Land cover with sites of factories, warehouses, and energy facilities (%)	
Park	Land cover with public green spaces for recreation and relaxation (%)	
Quarry	Land cover with sites for extracting stone, sand, gravel or clay (%)	
Retail	Land cover with land for shops, malls and supermarkets (%)	
Scrub	Land cover with bushy vegetation; transitional between meadow and forest (%)	
Orchard	Land cover with area planted with fruit trees, e.g., apples and cherries (%)	
Allotments	Land cover with divided garden plots for private cultivation (%)	
Heath	Land cover with dry, nutrient‐poor shrubland dominated by heath plants (%)	
Military	Land cover with area used for defence, training or military bases (%)	
Recreation ground	Land cover with public sports and play areas, e.g., fields and skateparks (%)	
Vineyard	Land cover with systematically planted area for grape production (%)	
Riverbank	Land cover with narrow strips along rivers, often vegetated slopes (%)	
Water	Land cover with general surface water bodies: lakes, ponds or rivers (%)	
Reservoir	Land cover with artificial water storage like dams or ponds (%)	
Wetland	Land cover with water‐saturated land, e.g., swamps and marshes (%)	
Altitude	Grid of elevation (m above sea level)	NASA Shuttle Radar Topography Mission
Livestock variables		Thünen Agraratlas, Neuenfeldt et al.[56]
Cattle	Number of cattle per km^2^	
Pigs	Number of pigs per km^2^	
Chickens	Number of chickens in 1000 individuals per km^2^	
Equines	Number of equines per km^2^	
Sheep	Number of sheep per km^2^	
Ruminants	Livestock units of ruminants per km^2^	
Game variables		Thünen Institute, Waldatlas [57]
Roe deer	Mean number of hunted roe deer per 100 ha and year	
Red deer	Mean number of hunted red deer per 100 ha and year	
Wild boar	Mean number of hunted wild boar per 100 ha and year	
Mouflons	Mean number of hunted mouflons per 100 ha and year	
Fallow deer	Mean number of hunted fallow deer per 100 ha and year	

Rasterised climate data for 2017 at 1 km resolution were obtained from the German Climate Data Center (DWD Climate Data Center (CDC): Annual Grids, version v1.0). Thirteen grids with data on temperature, precipitation, humidity and vegetation parameters were selected based on biologically plausible links to SBV transmission (Table 1). In addition, grid data of 19 bioclimatic variables were retrieved from the WorldClim database at approximately 1 km resolution, based on historical monthly temperature and precipitation data from 1970 to 2000 ([55]; accessed February 11, 2025; https://www.worldclim.org/data/bioclim.html). These variables are widely used in species distribution modelling and are included here to capture ecologically relevant climate gradients (Table 1). Raster data of annual mean wind power density in 50 m height in Germany with a resolution of approximately 200 m were retrieved from the Global Wind Atlas (https://globalwindatlas.info/en/download/gis-files, accessed December 3, 2024; [54]). Elevation data for Germany, as a raster with 100 m resolution, was obtained from the NASA Shuttle Radar Topography Mission (SRTM_GL3, 2013. Shuttle Radar Topography Mission (SRTM) Global. Distributed by OpenTopography. https://doi.org/10.5069/G9445JDF, accessed December 10, 2024). To spatially relate environmental raster data to sheep seroprevalence, a 5 km radius was drawn around each flock’s coordinates, defining an approximately 79 km^2^ buffer area. The radius reflects the limited flight range of the *Culicoides* spp., which typically do not disperse more than 5 km from their breeding sites [64, 65]. Median values for each raster layer within each buffer were extracted and used in subsequent analyses.

Land use data were obtained as vector shapefiles from OpenStreetMap via Geofabrik (https://download.geofabrik.de/europe/germany.html, accessed July 16, 2025). Land cover was classified into 24 categories, including forest, meadow, nature reserve, residential, cemetery, commercial, farmland, farmyard, grass, industrial, park, quarry, retail, scrub, orchard, allotments, heath, military, recreation ground, vineyard, riverbank, water, reservoir and wetland. Precise definitions are listed in Table 1. To quantify land use composition, vector polygons were intersected with the 5 km sheep flock buffers, and the proportional area of each land use class within each buffer was computed.

Livestock census data (2016) on cattle, pigs, chickens, equines and sheep were obtained at municipality level from official German agricultural statistics ([56]; Thünen Institut, 2024). Counts were standardised to population densities (individuals per km^2^) for each species. Additionally, densities of domestic ruminants (cattle and sheep) were combined into a single livestock unit (LU) metric, where one cattle or 10 sheep per km^2^ equated to one LU/km^2^ [66]. No standardised population census exists for relevant wildlife species, so data on wildlife species were represented by average annual hunting bag statistics from 2013 to 2017. Species included roe deer (*Capreolus capreolus*), red deer (*Cervus elaphus*), wild boar (*Sus scrofa*), mouflon (*Ovis gmelini musimon*) and fallow deer (*Dama dama*). Data were sourced from the Thünen Institut’s ‘Waldatlas’ and expressed as individuals per 100 ha at the county level (https://gdi.thuenen.de/wo/ http://waldatlas/?workspace=waldatlas_wild&instanz=wo, accessed December 6, 2024).

The results of a previously conducted and published risk analysis showed no specific effects of management‐related factors on SBV seroprevalence in sheep, but strong effects of sex and age were observed [45]. Accordingly, two animal‐related variables (female proportion and mean age) were included in the subsequent variable selection procedure.

### 2.3. Spatial Risk Modelling

**Figure 2:**
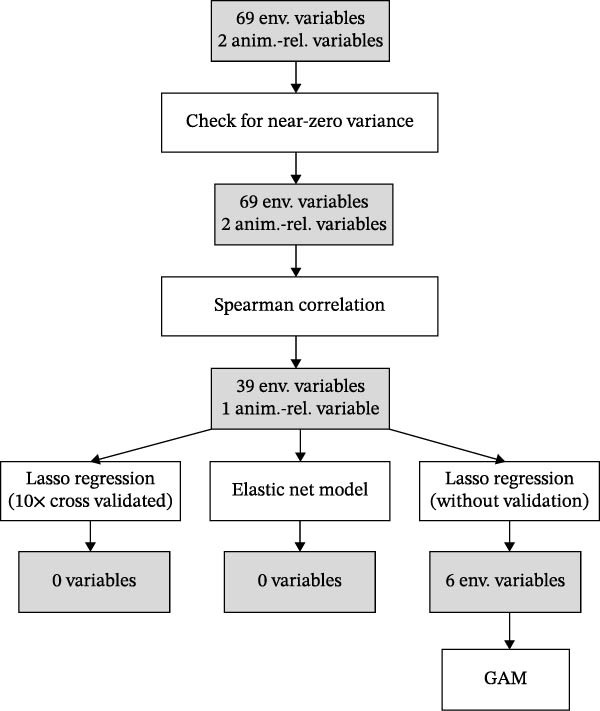
Flowchart illustrating the multi‐stage variable selection process from 69 environmental and two animal‐related variables, leading to the final set of predictors used in the generalised additive model (GAM).

Statistical analyses were conducted within the software R version 4.4.0 [59], using a general significance threshold of *α* = 0.05. Although quantitative antibody levels on individual animal level could provide additional insights into the intensity of exposure within flocks, spatial risk modelling investigated SBV seroprevalence data on flock level based on the binary serological outcome on individual animal level to prevent pseudo‐replication and to examine the effect of environmental factors at the location of each herd. The outcome variable hence represents the proportion of seropositive individuals within each flock. To identify the best explaining environmental and animal‐related predictors of SBV seroprevalence, a multi‐stage variable selection and modelling approach was applied, culminating in a generalised additive model (GAM). The overall workflow is summarised in Figure 2. Initially, all 69 environmental variables (Table 1), along with two animal‐related variables (female proportion, mean age), were screened for near‐zero variance using the ‘nearZeroVar()’ function from the R package ‘caret’ [67]. To ensure that only informative variables with sufficient variability remain for model fitting, variables with frequency ratios above 19 or less than 10% of unique values were removed. In succession, a pairwise Spearman correlation matrix was computed for all remaining candidate variables. Pairs of variables with an absolute correlation coefficient *r*_s_ ≥ 0.7 were considered highly collinear. For each highly correlated pair, only one variable based on an automated heuristic using the ‘findCorrelation()’ function from the ‘caret’ R package [67] was retained. To assess the remaining level of multicollinearity in the dataset, the condition number (*κ*) of the scaled correlation matrix was calculated. To further reduce dimensionality and identify the most influential predictors, we applied penalised regression models using the ‘glmnet’ package in R. A binomial Lasso regression (alpha = 1) with 10‐fold cross‐validation, and subsequently, an elastic net model (alpha = 0.5) as a more lenient alternative was fitted. Additionally, a full Lasso path without cross‐validation was fitted to examine variable entry order. From this, the top six predictors with the most frequent non‐zero coefficients across the Lasso path were selected for inclusion in the final GAM.

The explanatory spatial risk model was fitted as a GAM with a binomial error distribution and a logit link, using the ‘mgcv’ package. The model included the six selected predictors and a smooth term for geographic coordinates to account for spatial autocorrelation. Model performance was assessed using the deviance explained and the Akaike Information Criterion (AIC). For interpretability, odds ratios (ORs) and 95% confidence intervals were calculated from the parametric model terms.

### 2.4. Assessment of Spatial Autocorrelation

Spatial autocorrelation in farm‐level seroprevalence and model residuals was assessed using Moran’s *I*, determined utilising the ‘spdep’ R package [68]. Coordinates of farms were used to define spatial relationships based on either eight nearest neighbours or a 50 km distance threshold. The spatial weights were calculated to ensure that each farm accounts equally for its neighbours. Farms without neighbours were also included in the analysis. Global Moran’s *I* was applied to the seroprevalence values and to the deviance residuals from the GAM to indicate unexplained spatial structure by significant residual autocorrelation. Local indicators of spatial association (LISA; [69]) were computed using local Moran’s *I* to detect spatial clusters. Both, the local *I* values and their respective significance (*p* < 0.05) was mapped.

### 2.5. Prediction of SBV Seroprevalence

To spatially map predicted SBV seroprevalence in sheep flocks across the five federal states included in the study, a predictive GAM (binomial error distribution, logit link) was implemented over a hexagonal grid. The grid was generated with a cell size of 10 km. Only centroids of hexagons fully contained within the study area boundary were retained. A 5 km buffer radius was created around each centroid. Environmental variables for each centroid buffer were extracted using the same feature engineering pipeline as applied to the sampled sheep flocks, including summarising land use, climate, wind, elevation and host density variables. Farm‐specific animal‐related predictors (such as mean age or female sex proportion) were excluded to enable extrapolation to unsampled locations. The resulting predictions were visualised as continuous spatial risk surface of estimated SBV seroprevalence for sheep flocks.

## 3. Results

### 3.1. Spatial Risk Modelling

During the variable selection process, none of the 69 environmental and two demographic variables were removed due to near‐zero variance. In the subsequent pairwise Spearman correlation screening, 30 environmental and one animal‐related (mean age) highly correlated variables (*r*_s_ ≥ 0.7) were excluded using automated selection. A condition number (*κ*) of 330.8 was calculated for the reduced predictor matrix, indicating strong remaining multicollinearity among the selected variables. Penalised regression techniques and generalised additive modelling were subsequently used to help mitigate these effects. Lasso regression with tenfold cross‐validation retained no variables (all coefficients shrunk to zero), and elastic net regression (*α* = 0.5) also failed to retain any predictors. However, a relaxed Lasso model (without cross‐validation) identified six environmental variables that consistently exhibited non‐zero coefficients: BIO8 (mean temperature of the wettest quarter), cattle, sheep, vineyard, park and nature reserve. An overview of the variable selection process is given in Figure 2 and Table 2.

**Table 2:** Overview of variable reduction during the selection process preceding generalised additive model fitting.

Initial set of variables	Variables retained after Spearman correlation analysis	Variables selected by Lasso regression without cross validation
13 climate variables	Sunshine duration	
	Drought index	
	End of vegetation	
Wind power density		
19 bioclimatic variables	BIO1	
	BIO3	
	BIO8	BIO8
	BIO15	
24 land use variables	Forest	
	Meadow	
	Nature reserve	Nature reserve
	Commercial	
	Farmland	
	Farmyard	
	Grass	
	Industrial	
	Park	Park
	Quarry	
	Retail	
	Scrub	
	Orchard	
	Allotments	
	Heath	
	Military	
	Recreation ground	
	Vineyard	Vineyard
	Riverbank	
	Water	
	Reservoir	
	Wetland	
Altitude		
Six livestock variables	Cattle	Cattle
	Pigs	
	Chickens	
	Equines	
	Sheep	Sheep
Five game variables	Roe deer	
	Red deer	
	Wild boar	
	Mouflons	
	Fallow deer	
Female proportion mean age	Female proportion	

The final GAM incorporating these six selected predictors (Table 3) along with a spatial smoother for coordinates, explained 50.6% of the deviance and yielded an adjusted *R* ^2^ of 0.182. The model’s AIC was 486.33. Three of the parametric predictors showed statistically significant association with SBV seroprevalence in the sampled sheep flocks (Figure 3): Cattle density was positively associated with higher seroprevalence (OR = 1.01, 95% CI: 1.01–1.01, *p* < 0.001). BIO8 exhibited a negative association (mean temperature of the wettest quarter; OR = 0.95, 95% CI: 0.91–0.99, *p* = 0.021). Nature reserve coverage was also negatively associated with SBV seroprevalence (OR = 0.13, 95% CI: 0.02–0.67, *p* = 0.015). Other predictors (vineyard, park, and sheep density) were not statistically significant (*p* > 0.5). The spatial smoother for longitude and latitude was highly significant (*p* < 0.001), suggesting residual spatial heterogeneity not fully explained by the included predictors.

Figure 3Spatial distribution of the three predictor variables significantly associated with Schmallenberg virus seroprevalence in domestic sheep flocks. Each hexagonal grid cell has a diameter of 10 km. (a) Cattle population density (risk factor). (b) Mean temperature of the wettest quarter (BIO8, protective factor). (c) Land use proportion of nature reserve area (protective factor).

**Figure:**
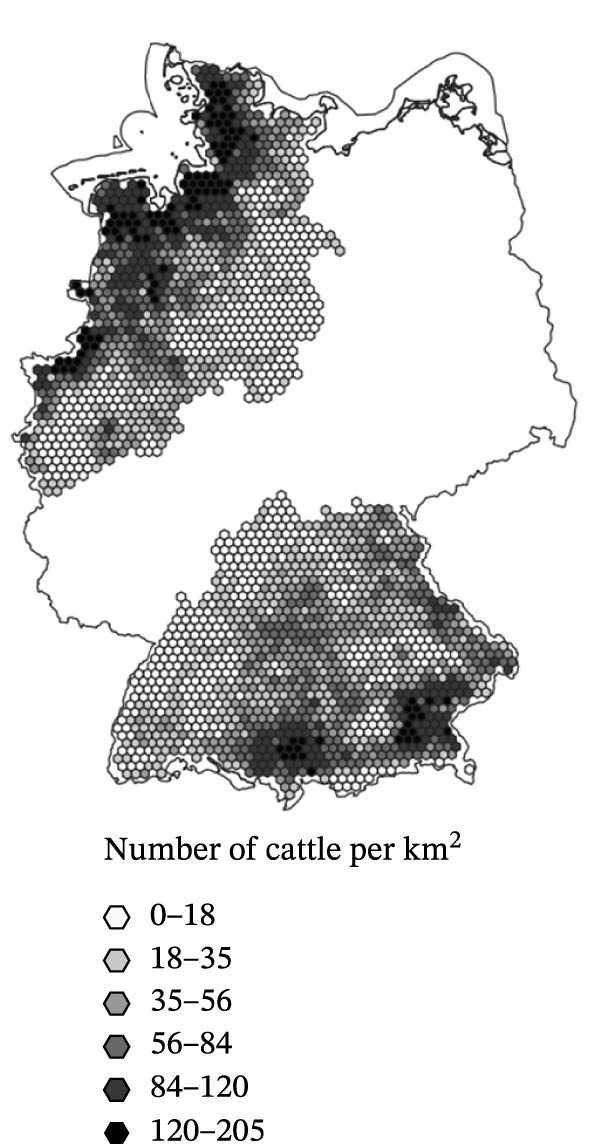
(a)

**Figure:**
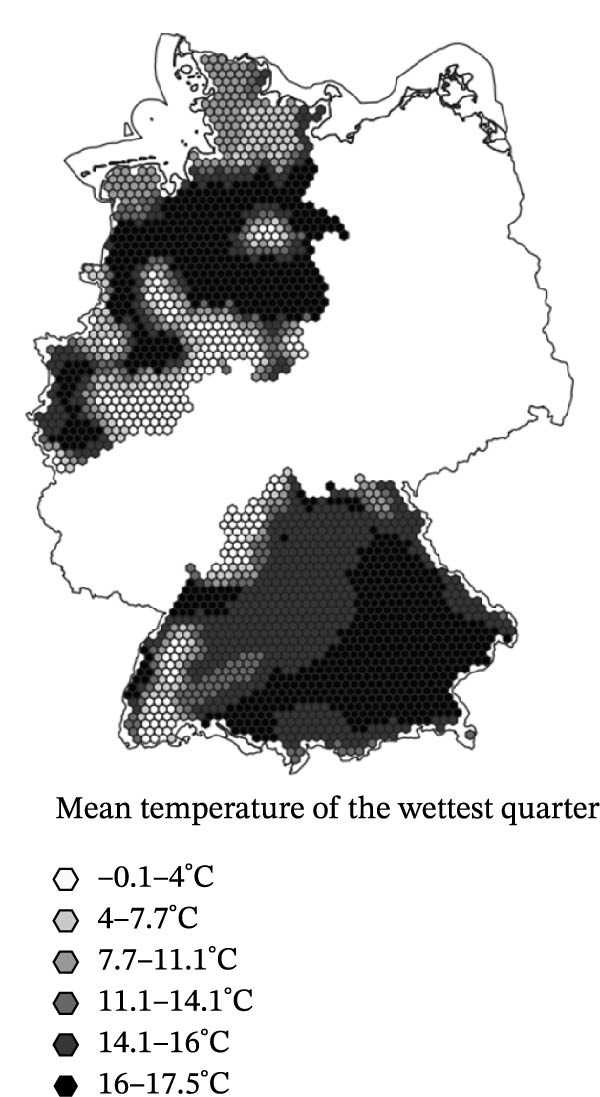
(b)

**Figure:**
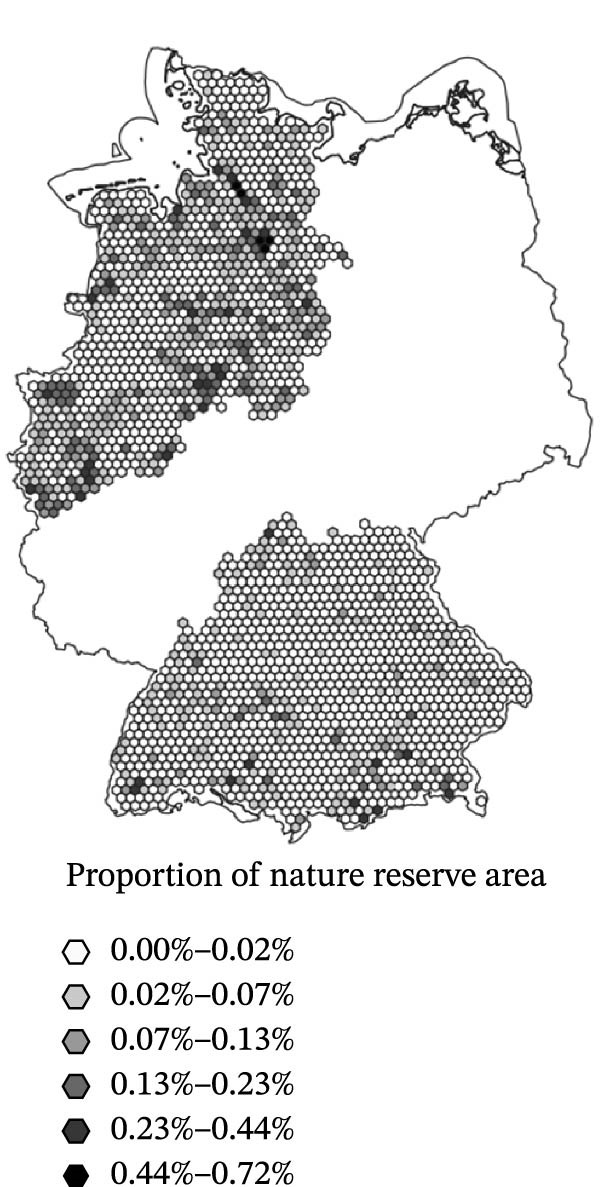
(c)

**Table 3:** Results of the generalised spatial additive risk modelling for the exposure of sheep to the Schmallenberg virus.

Variable	Odds ratio	95% CI (lower)	95% CI (upper)	*p*‐Value
Intercept	1.97	1.11	3.51	**0.02030**
BIO8	0.95	0.91	0.99	**0.02050**
Cattle	1.01	1.01	1.01	**0.00002**
Vineyard	10.36	0.004	24,435.56	0.55510
Nature reserve	0.13	0.02	0.67	**0.01520**
Park	2.06E+09	0.37	1.16E+19	0.06120
Sheep	1	0.996	1.01	0.64090

*Note:* *p*‐Values < 0.05 are indicated in bold. BIO8: mean temperature of the wettest quarter of the year.

### 3.2. Assessment of Spatial Autocorrelation

Using the eight nearest neighbours, no significant spatial autocorrelation was detected in the observed farm‐level seroprevalence values (Moran’s *I* = –0.061, *p* = 0.814) or in the GAM residuals (Moran’s *I* = –0.131, *p* = 0.987). Similarly, applying a 50 km distance‐based neighbourhood also revealed no significant global spatial autocorrelation in seroprevalence values (Moran’s *I* = 0.0004, *p* = 0.448). Finally, local Moran’s *I* (LISA) analysis showed that most sheep flocks exhibited non‐significant local Moran’s *I* values (*p*  > 0.05), and no pronounced spatial clusters were identified (Figure 4).

Figure 4Local Moran’s *I* analysis of SBV seroprevalence across 70 sampled sheep flocks based on the eight nearest neighbours. (a) Local Moran’s *I* values indicating the degree of spatial autocorrelation. (b) Corresponding *p*‐values (*p*  < 0.05) indicating statistical significance. The analysis revealed no strong spatial clusters; most farms exhibited non‐significant local statistics.

**Figure:**
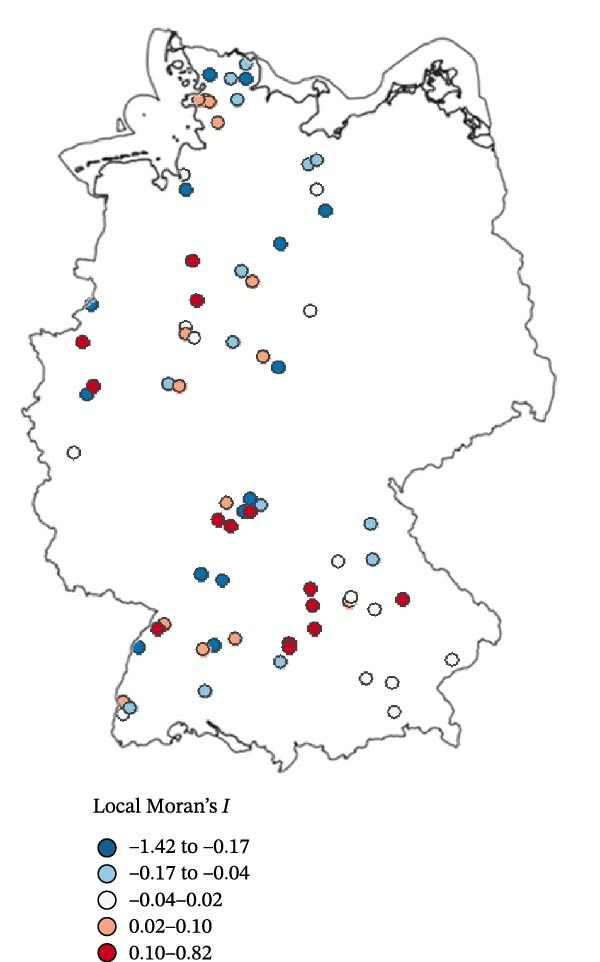
(a)

**Figure:**
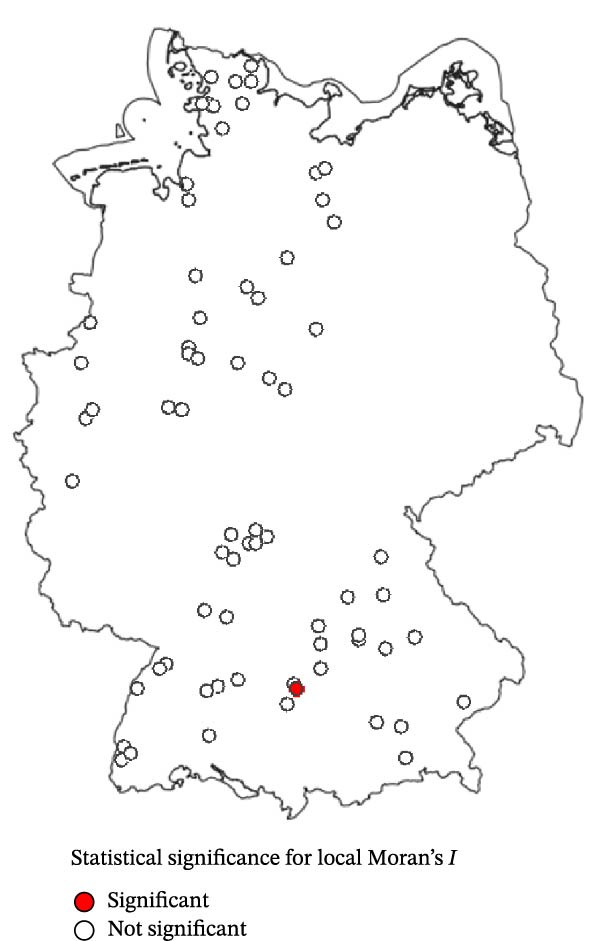
(b)

### 3.3. Prediction of SBV Seroprevalence

To generate spatial predictions, the previously selected six environmental variables (BIO8, cattle, sheep, vineyard, park and nature reserve) and a spatial smoother were included in the predictive GAM, which was applied to a hexagonal grid of 2102 centroids across the five federal states in the study area. Since no animal‐related variables were selected for the final GAM, the full set of six environmental variables was used in the predictive model as well, resulting in the same AIC as the full explanatory model (AIC = 486.33). Predicted SBV seroprevalence across the grid ranged from 25.45% to 97.37%, with an average prediction close to the observed mean of 60.4%. The predicted risk surface (Figure 5) displays a largely homogeneous distribution of SBV seroprevalence in sheep flocks (exposure risk) across the mapped study area. Slightly elevated risk estimates were observed in the Bavarian–Czech border region, the Black Forest and the North Sea coastal area. Conversely, Central South Germany, Southwestern Lower Saxony and areas around the Baltic Sea coast exhibited slightly lower predicted seroprevalence rates compared to the mean.

Figure 5Predicted Schmallenberg virus (SBV) seroprevalence in sheep flocks across the five surveyed German federal states. Predictions are based on a generalised additive model (GAM) fitted across a 10 km hexagonal grid. (a) Map of spatial variation in predicted SBV seroprevalence in sheep flocks beyond known predictors. The smoothed surface highlights areas with higher (red) or lower (blue) predicted risk than expected based on the variables included in the fitted GAM. (b) Map of model‐predicted Schmallenberg virus (SBV) seroprevalence in sheep flocks. Grid cell colour grading show predicted seroprevalence (blue = low and yellow = high). Dots represent sampled sheep flocks, coloured by observed within‐flock SBV seroprevalence.

**Figure:**
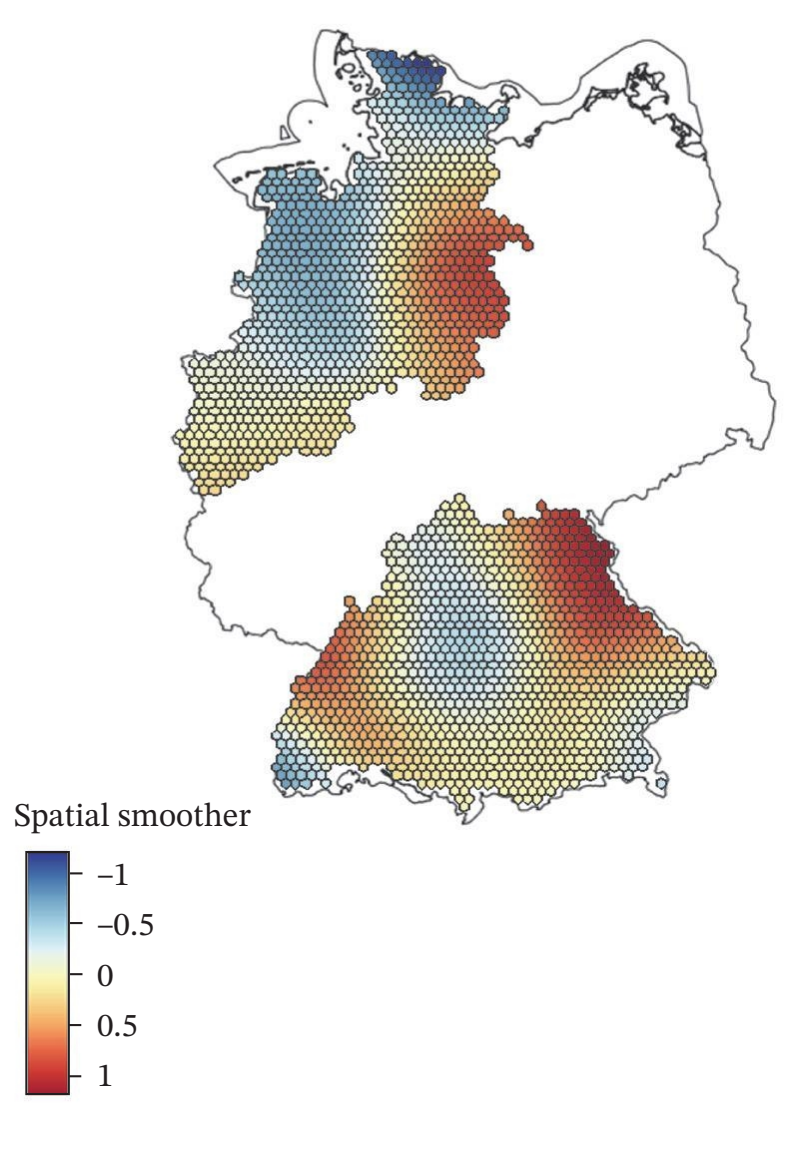
(a)

**Figure:**
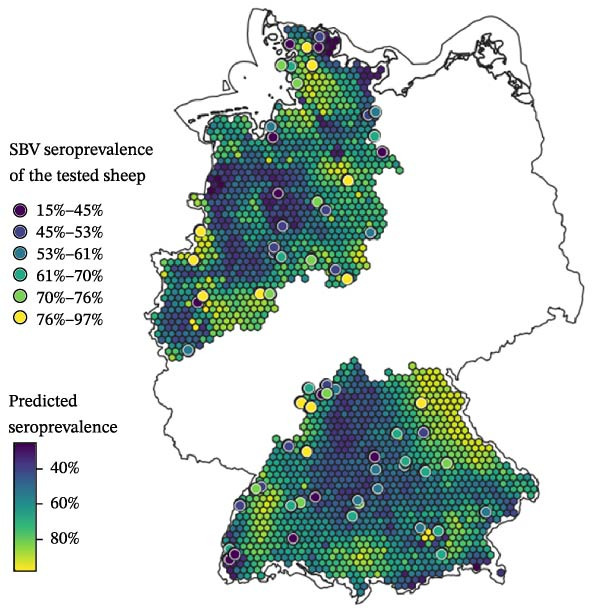
(b)

## 4. Discussion

### 4.1. Climate Effects

Although previous research highlights the influence of climate on *Culicoides* vector ecology and virus transmission [38, 39, 70, 71], most of the climate parameters investigated in this study did not significantly explain SBV seroprevalence in sheep flocks. A positive association with warm and humid climatic conditions was initially expected but not supported. Notably, none of the variables related to precipitation (precipitation, BIO12–BIO19), soil humidity (drought index), or temperature extremes (frost, hot and summer days) were significant. Contrary to initial hypotheses, BIO8 (mean temperature of the wettest quarter) showed a negative association with SBV seroprevalence (Table 3 and Figure 3b). The effect size of BIO8 as a protective factor (OR = 0.95) refers to a 1°C increase in the mean temperature of the wettest quarter. Based on an average SBV seroprevalence of 60.7 % and a mean temperature of 11.44°C, the expected prevalence at the extreme sites in the dataset is approximately 72.1 % at 1.38°C (most precipitation in winter) and 53.3 % at 17.37°C (most precipitation in summer). These findings suggest that seasonality of precipitation is more relevant for a successful vector function than the overall amount of rain per year (factor precipitation). High rainfall during the midge activity period in summer may inhibit transmission. Flight activity of the midges might be impaired and, possibly even more relevant, excessive saturation of the midge breeding substrates with water may disrupt larval development. While some *Culicoides* species depend on small accumulations of rainwater for breeding [27], the species of the *Culicoides obsoletus* complex, which serve as main vectors for SBV in central Europe, thrive in moist soil, manure, slurry and farm environments. The lack of effects from surface water variables (riverbank, water, reservoir and wetland), and the positive association with cattle density reinforce the idea that farm‐level conditions, rather than broad climate patterns, are more relevant to vector habitat suitability and transmission risk of some *Culicoides*‐borne diseases [36, 37].

The role of wind remains unclear. Although strong winds, like heavy precipitation [4, 72, 73], are known to impair midge flight activity [74, 75], wind can also facilitate long‐distance vector dispersal [4, 27, 76]. Wind power density had no detectable effect here, possibly due to opposing mechanisms masking each other or limitations in the spatial resolution of wind data.

Seasonal temperature thresholds, such as 10°C for midge activity, were hypothesised to influence SBV seroprevalence in sheep flocks, especially during spring and autumn. However, variables describing recurring, temperature dependent seasonal events (onset of vegetation, apple flowering and end of vegetation) were non‐significant. This may reflect a mismatch between vegetation‐based and vector‐based temperature thresholds or a lack of fine‐scale temperature data. A more informative metric might have been the number of days with temperatures above 10°C, but such high‐resolution grid data were unavailable.

### 4.2. Land Use and Topography

Although no specific land use effects were anticipated prior to the analysis, some landscapes might be related with higher population densities of grazing ruminants serving as hosts for *Culicoides* spp. or link with microclimates [77] and breeding habitats of the midges in moist organic‐rich topsoil layers [30]. Here it was found that nature reserve coverage showed a significant protective effect on SBV higher seroprevalence in sheep flocks (Table 3 and Figure 3b). The effect could be related to nature reserve areas serving as habitats for predators of *Culicoides* species, including bats and insectivorous birds, which may regulate the population density of the insects. Overall, the observation is, however, unlikely to result from consistent environmental conditions within reserves, as they encompass heterogeneous landscapes. Instead, the absence of intensive agriculture activities, especially manure application, likely reduces breeding sites for SBV‐relevant *Culicoides* vectors. This interpretation aligns with the positive effect of cattle density, which is often linked to manure‐rich environments, favourable for vector proliferation [36, 37].

Other land use variables did not show significant effects. This may reflect limitations in the way land cover was quantified (just considering coverage proportions without spatial structure). Vector breeding is influenced not only by habitat types but also by their spatial configuration and microhabitat features (canopy height and soil type), which were not further characterised here. As Kirkeby et al. [78] recommend, incorporating variables like edaphic biomass or leaf area indices would likely improve models of *Culicoides* vector habitat suitability. This is, however, beyond the scope of this study.

No significant effect of altitude was observed, likely due to the narrow altitude range in the sampled sheep flocks (0–578 m, except for one at 910 m; mean = 224.7 masl, *σ* = 181.1 masl). By contrast, studies in more mountainous regions of Spain report reduced SBV detection rates at higher elevations over 400 m [39]. This was explained by the biology of the midges, which prefer different altitudes depending on the species [39, 79]. Although sporadic transmission events are documented [80], altitudes of over 2000 m above sea level are regarded as the limit. Given the predominant lowland setting of sheep flocks in this study, altitude may not have been a limiting factor. Interestingly, for the single sheep flock at higher altitude a lower seroprevalence was observed at 40.0%, which was significantly below the within‐herd average of 60.7%.

### 4.3. Host Availability

As expected, cattle density was significantly associated with SBV seroprevalence in the investigated sheep flocks. The calculated OR of 1.01 refers to an increase in cattle population density of one individual animal per km^2^. Assuming an average SBV seroprevalence in sheep of 60.7 % and a mean cattle population density of 38.66 individuals/km^2^, the expected prevalence at the extreme sites in the dataset is approximately 51.3 % at the site with lowest (0.001 individual per km^2^) and 91.9 % at the site with highest cattle density (239.9 individuals per km^2^). In contrast, sheep density had no effect. Cattle are known to develop higher SBV RNA loads than sheep during infection [17] possibly making them more effective amplifying hosts. Furthermore, *Culicoides* feeding preferences strongly favour cattle. Studies in Denmark showed that up to 77% of blood meals were taken from cattle, compared to just 0.4% from sheep [81]. This preference likely facilitates intra‐cattle transmission and subsequent spillover to co‐located sheep, increasing infection pressure without requiring high sheep densities. A similar effect was found for goats co‐located with cattle in the related study on husbandry‐related SBV risk factors [45].

Environmental conditions on cattle farms, such as large quantities of manure and maize silage use, create ideal *Culicoides obsoletus* complex breeding sites [27, 82]. Other livestock species such as pigs and poultry also produce manure but appear to play a limited role in SBV epidemiology. This may relate to lower host attractiveness or vector preference, as pigs and chickens are rarely targeted by relevant *Culicoides* species [83, 84].

### 4.4. Prediction of SBV Seroprevalence

The predicted SBV seroprevalence risk surface (Figure 5b) revealed a largely homogeneous distribution pattern across the five surveyed federal states with only modest spatial heterogeneity. This observation aligns with the absence of global spatial autocorrelation or pronounced local spatial clustering of SBV seroprevalence in the examined sheep flocks (Figure 4), indicating an unstructured and homogeneously distributed risk for SBV exposure in sheep flocks. Slightly elevated risk estimates were observed in the Bavarian–Czech border region, the Black Forest and the North Sea coastal area, while Central South Germany, Southwestern Lower Saxony and areas around the Baltic Sea showed lower values. While the included variables (BIO8, cattle, sheep, vineyard, park and nature reserve) captured some environmental variation, the moderate adjusted *R* ^2^ (0.182) and the significant spatial smoother indicate that key drivers remain unmeasured. These could include microclimatic conditions, fine‐scale vector dynamics, farm management practices or host immunity profiles, for which no data are available to us. As such, while the model provides useful approximation of regional exposure patterns, predictive accuracy may improve with finer‐resolution data and additional explanatory variables.

### 4.5. Limitations and Conclusions

This study aimed to identify environmental risk factors for SBV seroprevalence in sheep flocks in a largely endemic setting in Germany (autumn 2017 to spring 2018), several years after the virus’s initial emergence. The studied animals hence likely experienced lifetime exposure, enabling the detection of long‐term risk patterns. Such data might help to detect subtle differences in transmission pressure that remain obscured when using a simple positive/negative classification. However, despite widespread infection (mean seroprevalence 60.7%), no strong environmental hotspots were identified. This may be due to the relatively homogeneous landscape and climate in the study area, limiting environmental contrast. In more topographically or climatically diverse settings, such as landscapes in France or Spain, stronger associations between SBV and environmental risk factors may have been observed. It should also be noted that the cyclical pattern of SBV exposure [24] could not be captured by the cross‐sectional data used in this study. Investigations covering longer time periods would be required to address this aspect, which was, however, beyond the scope of the present study. Furthermore, many of our hypotheses implicitly assumed a direct link between *Culicoides* abundance and SBV seroprevalence, yet no vector data were available to validate this assumption. Unlike BTV, for which vector‐abundance (*C. imicola*) has been directly linked to BTV outbreaks in Southern Europe [85], similar data for SBV are lacking, particularly in Central Europe.

Nonetheless, the consistent positive association with cattle density underscores the importance of amplifying hosts and farm‐level conditions in shaping SBV risk. This suggests that cattle should be a particular focus of SBV monitoring efforts, and vector control strategies, whereby manure handling in particular may offer practical SBV mitigation options [27]. While landscape design has been proposed for SBV/BTV control [27], our findings suggest such strategies may have limited impact in temperate, homogenous environments like Germany.

In the light of ongoing climate change, with increasing temperatures and extended vector activity periods, SBV transmission dynamics may evolve. Although precise effects remain unpredictable, warming trends could widen the seasonal window for virus circulation, particularly given the cyclic nature of SBV outbreaks [24].

Despite similarities in transmission ecology between SBV and BTV [27, 86], the viruses differ in key virological properties. SBV’s short latent period, brief viraemia and rapid replication may allow for highly efficient spread, even more rapidly than BTV‐8 [6, 38, 87]. As such, findings from this study should not be directly extrapolated to BTV, and pathogen‐specific models, monitoring approaches and classification strategies remain essential. This highlights that Central Europe is still developing its understanding and preparedness for vector‐borne viruses.

In conclusion, while climatic and land use variables played a limited explanatory role in this study, the results highlight the vulnerability of German ruminant populations to *Culicoides*‐borne viruses, as recently demonstrated by the rapid spread of BTV serotype 3 across the country [88, 89]. Continued surveillance and modelling efforts, incorporating vector ecology, host distribution and farm practices, are essential for anticipating future outbreaks and informing control strategies.

## Author Contributions

Conceptualisation: Frederik Kiene, Martin Ganter and Benjamin U. Bauer. Methodology: Frederik Kiene, Hannes Bergmann and Benjamin U. Bauer. Formal analysis, visualisation: Frederik Kiene and Hannes Bergmann. Investigation: Frederik Kiene and Benjamin U. Bauer. Resources: Martin Ganter. Data curation, writing – original draft: Frederik Kiene. Writing – review and editing: Martin Ganter, Hannes Bergmann and Benjamin U. Bauer. Supervision, project administration: Benjamin U. Bauer.

## Funding

Open Access funding enabled and organized by Projekt DEAL.

## Disclosure

All authors have read and agreed to the published version of the manuscript.

## Ethics Statement

Serum samples from sheep were originally collected as part of a previous study [52, 90], which was approved by the federal state governments of Schleswig‐Holstein (V 242‐5191/2018), Lower Saxony (AZ 33.8‐42502‐05‐17A211), North Rhine‐Westphalia (81‐02.05.40.18.015), Baden‐Wuerttemberg (AZ 35‐9185.82/0351, AZ 35‐9185.82/D‐18/01, AZ 35‐9185.82/A‐1/18 and AZ 35/9185.82/Ganter 18.01.2018) and Bavaria (RUF‐55.2.2‐2532‐2‐651‐5 and ROB‐55.2‐2532.Vet_03‐18‐10). The study was conducted in accordance with the ethical standards determined by the German Animal Welfare Legislation, the EU Directive 2010/63/EU on the protection of animals used for scientific purposes and the ARRIVE guidelines. Protocols to ensure full compliance with national regulations on animal care and handling were implemented. All procedures were carried out with the highest ethical consideration and adherence to established standards.

## Conflicts of Interest

The authors declare no conflicts of interest.

## Data Availability

The data are available upon request.
